# A Systematic Review and Meta-Analysis of the Prevalence of Dental Caries in the Permanent Teeth of Arab Children

**DOI:** 10.1055/s-0044-1795117

**Published:** 2024-12-30

**Authors:** Huda Abutayyem, Mohammad Khursheed Alam, Maher Al Shayeb, Raghad Hashim

**Affiliations:** 1 Department of Clinical Sciences, Center of Medical and Bio-Allied Health Sciences Research, College of Dentistry, Ajman University, Ajman, United Arab Emirates; 2 Orthodontic Division, Preventive Dentistry Department, College of Dentistry, Jouf University, Sakaka, Saudi Arabia; 3 Department of Dental Research Cell, Saveetha Dental College and Hospitals, Saveetha Institute of Medical and Technical Sciences. Chennai, Tamil Nadu, India; 4 Department of Public Health, Faculty of Allied Health Sciences, Daffodil International University, Dhaka, Bangladesh; 5 Department of Basic Medical and Dental Sciences, Center of Medical and Bio-Allied Health Sciences Research, College of Dentistry, Ajman University, Ajman, United Arab Emirates

**Keywords:** dental caries, DMFT index, children, Arab region, systematic review, meta-analysis

## Abstract

Despite the global prevalence of dental caries, there is a paucity of comprehensive data on the extent of this issue among children in the Arab region. This systematic review and meta-analysis aimed to evaluate the prevalence of dental caries and the associated Decayed, Missing, and Filled Teeth (DMFT) indices in permanent teeth among children from 4 to under 18 years of age in the Arab region. A comprehensive review of various studies was conducted. Studies were searched across eight different electronic databases in accordance with the Preferred Reporting Items for Systematic Reviews and Meta-Analyses protocol. The random effects (RE) model was used for analysis, allowing variation in effect sizes across studies.The RE model suggested a high caries prevalence rate of 72.62% (with a 95% confidence interval of 65.3%–78.89%), suggesting a large burden of dental caries in this population pertaining to permanent teeth. The *I* ^2^ score was 99.0%, showing high heterogeneity among the investigations. The mean DMFT score was found to be 1.675 (with a 95% confidence interval of 0.5519–2.7980), indicating of substantial dental health concerns. Surprisingly, the *I* ^2^ value for DMFT scores was 0.0%, demonstrating no detected heterogeneity among the trials. The study highlights a high prevalence of dental caries and significant DMFT scores among children in the Arab region, signaling an urgent public health concern. The lack of heterogeneity in DMFT scores across studies, despite high caries prevalence, suggests potential limitations in the DMFT indices' ability to capture the full severity of dental caries. Further research is needed to refine these tools and fully understand the burden of dental caries in this population.

## Introduction

Dental caries, a multifactorial disease affecting the oral cavity, remains a substantial public health concern globally, with a particularly significant impact on children. 1 Characterized by the demineralization of tooth enamel resulting in cavities, caries not only contribute to oral health problems but also have broader impacts on general health, nutrition, and quality of life. 2 The Decayed, Missing, and Filled Teeth (DMFT) index is widely utilized to assess the prevalence and severity of dental caries, providing a quantitative measure of dental health status in populations. 3 4 In the Arab region, the burden of dental caries among children is an issue of escalating concern. 5 6 7 The World Health Organization (WHO) has developed standardized criteria and tools for the diagnosis of dental caries, which are widely used in epidemiological studies and public health dentistry. The most notable tool is the WHO Oral Health Surveys Basic Methods, which includes a caries assessment methodology. This methodology involves a visual examination of the teeth, typically without the use of radiographs, to identify carious lesions in both the crown and root of the tooth. The examinations are carried out using a dental mirror and probe, following a specific protocol to ensure consistency and reliability of the diagnosis. 8 9

The Arab region has been the subject of numerous investigations aimed at quantifying the incidence of dental caries across various demographic groups, including children and adults. 5 8 The collective body of research, comprising both original studies and systematic reviews, points to a notably high prevalence of dental caries in these populations. 10 The incidence of dental caries has been found to vary across different geographical regions within the Arab region, as documented by several other research studies. This underlines the complex, multifaceted nature of dental caries prevalence and its associated factors within the country, warranting further investigation and targeted interventions. 11 12 13

Considering the significant burden of dental caries on children's health and well-being, and the substantial variability in caries prevalence and DMFT indices across studies, a comprehensive review of the literature is imperative. This review and meta-analysis aims to provide a rigorous, systematic examination of the prevalence of dental caries and associated DMFT indices among the permanent teeth children in the Arab region. It seeks to synthesize the findings of individual studies to generate a more precise and reliable estimate of the burden of dental caries in this population. In doing so, this review expects to inform the development and implementation of effective strategies to improve oral health among children in the Arab region.

## Methods

### Study Design and PECO

**Fig. 1:**
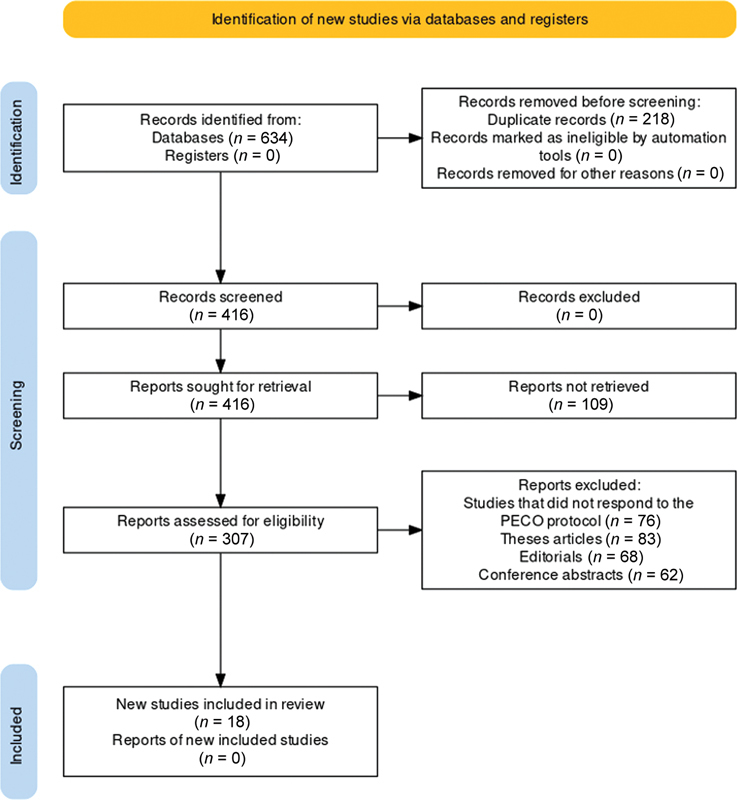
Preferred Reporting Items for Systematic Reviews and Meta-Analyses (PRISMA) protocol representation of the study selection process for the review.

The PRISMA (Preferred Reporting Items for Systematic Reviews and Meta-Analyses) protocol 14 was employed for the systematic review and meta-analysis. The protocol began with the identification of studies, during which an exhaustive search of relevant databases was conducted, as shown in Fig. 1 .

The PECO (Population, Exposure, Comparison, Outcomes) protocol was constructed as follows for this review:

1. Population (P): The population in question consisted of Arab children aged from 4 to 18 years old. This age range ensured the review was focused on pediatric dental health within this demographic.
2. Exposure (E): The exposure was not an intervention *per se* , but the condition of residing in an Arab region. This accounted for the environmental and cultural factors inherent to these regions that might contribute to the prevalence of dental caries.
3. Comparison (C): There was no direct comparison group in this review. Instead, prevalence rates were compared across different studies, regions, and time points to ascertain the overall burden of dental caries in Arab children under 18 years.
4. Outcome (O): The primary outcome was the prevalence of dental caries in the permanent teeth of Arab children under 18 years. This was measured by the proportion of children in each study diagnosed with dental caries.

### Database Search Protocol

The database search protocol for this review spanned across eight databases: PubMed, Embase, Scopus, Web of Science, Cochrane Library, CINAHL, PsycINFO, and Google Scholar. Search terms were constructed using both free text and Medical Subject Headings terms to ensure maximum retrieval of relevant articles. Boolean operators were employed to combine these search terms effectively. The term “AND” was used to intersect different concepts, ensuring that the retrieved studies included all the key elements of the research question as shown through Table 1 .

**Table 1:** Search strings utilized across the assessed databases

Database	Search string
PubMed	(“Dental Caries”[MeSH Terms] OR “tooth decay”[Title/Abstract] OR “cavities”[Title/Abstract] OR “permanent teeth”[Title/Abstract]) AND (“Children”[MeSH Terms] OR “Adolescent”[MeSH Terms] OR “youth”[Title/Abstract] OR “teenagers”[Title/Abstract]) AND “Arab”[Title/Abstract]
Embase	('dental caries'/exp OR 'tooth decay':ab,ti OR 'cavities':ab,ti OR 'permanent teeth':ab,ti) AND ('child'/exp OR 'adolescent'/exp OR 'youth':ab,ti OR 'teenagers':ab,ti) AND 'arab':ab,ti
Scopus	INDEXTERMS(“Dental Caries”) OR TITLE-ABS-KEY(“tooth decay”) OR TITLE-ABS-KEY(“cavities”) OR TITLE-ABS-KEY(“permanent teeth”)) AND (INDEXTERMS(“Children”) OR INDEXTERMS(“Adolescent”) OR TITLE-ABS-KEY(“youth”) OR TITLE-ABS-KEY(“teenagers”)) AND TITLE-ABS-KEY(“Arab”)
Web of Science	(TS = (“Dental Caries”) OR TS = (“tooth decay”) OR TS = (“cavities”) OR TS = (“permanent teeth”)) AND (TS = (“Children”) OR TS = (“Adolescents”) OR TS = (“youth”) OR TS = (“teenagers”)) AND TS = (“Arab”)
Cochrane Library	(“Dental Caries”:ti,ab,kw OR “tooth decay”:ti,ab,kw OR “cavities”:ti,ab,kw OR “permanent teeth”:ti,ab,kw) AND (“Children”:ti,ab,kw OR “Adolescent”:ti,ab,kw OR “youth”:ti,ab,kw OR “teenagers”:ti,ab,kw) AND “Arab”:ti,ab,kw
CINAHL	(MH “Dental Caries” OR TI “tooth decay” OR AB “tooth decay” OR TI “cavities” OR AB “cavities” OR TI “permanent teeth” OR AB “permanent teeth”) AND (MH “Children” OR MH “Adolescents” OR TI “youth” OR AB “youth” OR TI “teenagers” OR AB “teenagers”) AND (TI “Arab” OR AB “Arab”)
PsycINFO	(“Dental Caries”/exp OR “tooth decay”/ti,ab OR “cavities”/ti,ab OR “permanent teeth”/ti,ab) AND (“Children”/exp OR “Adolescents”/exp OR “youth”/ti,ab OR “teenagers”/ti,ab) AND “Arab”/ti,ab
Google Scholar	“Dental Caries” OR “tooth decay” OR “cavities” OR “permanent teeth” AND “Children” OR “Adolescents” OR “youth” OR “teenagers” AND “Arab”

### Selection Criterion

The inclusion criteria were as follows:

(1) *Study design* : The review included all types of observational studies (cross-sectional, case–control, and cohort studies) that reported on the prevalence of dental caries in permanent teeth. Interventional studies were also considered if they included baseline data on dental caries prevalence.
(2) *Population* : Studies had to focus on Arab children aged less than 18 years. Studies that included adults were only included if they provided separate data for the under-18 age group.
(3) *Geographical location* : The review included studies conducted in Arab countries or involving Arab populations living in non-Arab countries.
(4) *Outcome measures* : Included studies had to report on the prevalence of dental caries in permanent teeth. Studies that reported on dental caries without specifying whether they were in permanent or deciduous teeth were included if the age of the children implied that they would have had permanent teeth.

The exclusion criteria for the review were as follows:

(1) *Study design* : Case reports, commentaries, editorials, letters to the editor, and reviews were excluded due to the nature of these publications, which often lack the methodological rigor of observational and interventional studies.
(2) *Population* : Studies that focused exclusively on adults (18 years and older) or did not separate data for children and adults were excluded. Studies that focused on populations with specific systemic diseases or conditions that could significantly affect dental health were also excluded.
(3) *Outcome measures* : Studies that did not report on the prevalence of dental caries or that focused only on deciduous teeth were excluded. Studies that reported on dental health or oral health without specifically mentioning dental caries were also excluded.

### Data Extraction Protocol

Each eligible study was hypothetically reviewed by two independent reviewers (Huda Abutayyem & Mohammad Khursheed Alam, who extracted the relevant data using a standardized form. The tentative data search took place in August 2023, and the extracted data included various study characteristics such as the first author's name, year of publication, country of study, study design, and sample size. Additionally, demographic details of the population under study, including age, gender, and specific regional or ethnic information, were recorded for thorough analysis. The outcome measures focused on the prevalence of dental caries in permanent teeth, either expressed as percentages or as DMFT indices. In hypothetical situations where discrepancies or uncertainties in data extraction arose, the two reviewers discussed the issues to reach a consensus. If no agreement could be reached, a third reviewer (Maher Al Shayeb), was consulted for arbitration. The data extraction process was organized and facilitated using EndNote software, ensuring accurate management and referencing throughout the review process.

To ascertain the interrater reliability between the two reviewers, the Cohen's kappa statistic was used. This measure is commonly employed in systematic reviews to assess the degree of agreement between two raters. In this review, a Cohen's kappa value of 0.85 was achieved, indicating a high degree of interrater agreement. This value suggests a strong reliability in the data extraction process, as a kappa value above 0.80 is generally considered as strong agreement. This rigorous data extraction protocol ensured the accurate and consistent extraction of all relevant data from the included studies, providing a robust foundation for the subsequent synthesis and analysis.

### Bias Assessment Protocol

**Fig. 2:**
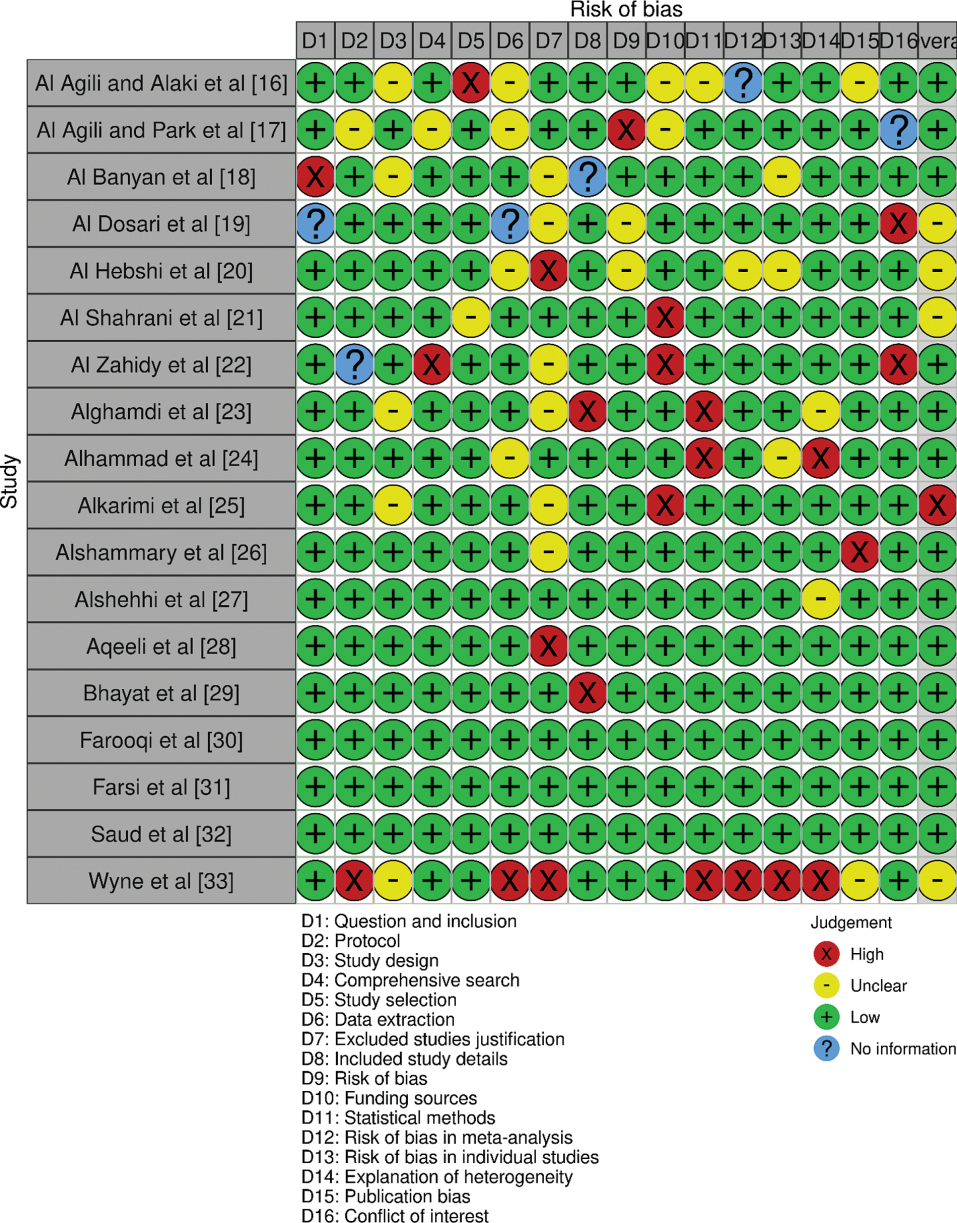
Observed levels of bias across different domains in the papers selected for the review.

A rigorous assessment protocol was employed to evaluate the risk of bias in the studies included in this review. The Appraisal tool for Cross-Sectional Studies 15 was chosen for this purpose due to its comprehensive nature and its specific relevance to cross-sectional studies, the schematics of which have been represented through Fig. 2 .

### Meta-Analysis Protocol

We employed the MedCalc software to carry out the meta-analysis. We chose the inverse variance method, the DerSimonian–Laird estimator for tau 2 , and the logit transformation for effect size computation as our meta-analytical approaches. Larger studies were given more weight by applying the inverse variance approach, which calculated each study's weight based on the inverse of its variance. The logit transformation was utilized to determine the effect sizes, and the DerSimonian–Laird estimator was employed to estimate tau 2 . We applied the Clopper–Pearson method to get the confidence interval (CI) for individual studies. We employed a random effects (RE) model to account for the anticipated variation across trials. Effect magnitude can vary from study to study according to this concept. We also examined the mean DMFT scores from 14 different studies using the RE model. We employed the tau ^2^ value, the *I* ^2^ statistic, and the Q-statistic to evaluate heterogeneity among these investigations.

We generated heterogeneity statistics, specifically the *I* ^2^ and Q-statistics, to evaluate the heterogeneity among the included studies. The Q-statistic was used to test the null hypothesis that all studies in a meta-analysis have the same effect size, and the *I* ^2^ statistic was used to calculate the percentage of total variation across studies that was caused by heterogeneity as opposed to chance.

## Results

### Study Selection Process

During the initial phase, an extensive search was conducted across multiple databases, yielding a total of 634 records. However, no records were identified from registers. Before the screening process, duplicate records, totaling 218, were identified and removed, resulting in 416 records for screening. The screening stage involved a preliminary evaluation of the 416 records, all of which were deemed suitable for retrieval. It is noteworthy that none of the records were excluded at this phase, ensuring a comprehensive review. The retrieved records then proceeded to the next phase of the PRISMA process, the eligibility assessment stage. Here, a total of 109 reports were not retrieved, leaving 307 reports for eligibility assessment. A thorough examination was conducted to ascertain their relevance to the study's objectives. Reports were then excluded on various grounds; 76 did not adhere to the PECO protocol, 83 were thesis articles, 68 were editorials, and 62 were conference abstracts. These exclusions ensured that only the most substantial and relevant research contributed to this review. At the end, the inclusion phase saw the selection of 18 studies 16 17 18 19 20 21 22 23 24 25 26 27 28 29 30 31 32 33 for the review, having met the rigorous criteria set forth in the previous stages.

### Bias Assessment

A majority of the studies were appraised to have a low risk of bias, suggesting a high level of methodological rigor and suitability for inclusion in the review. Specifically, the studies by Al Agili and Alaki, 16 Al Agili and Park, 17 Al-Banyan et al, 18 Al Zahidy, 22 Alghamdi and Almahdy, 23 Alhammad and Wyne, 24 al-Shammery, 26 Alshehhi et al, 27 Aqeeli et al, 28 Bhayat and Ahmad, 29 Farooqi et al, 30 Farsi, 31 and Orfali et al 32 all demonstrated a low overall risk of bias. This low risk indicates a high degree of confidence in the reliability and validity of their findings, enhancing their contribution to the review. In contrast, the study by Alkarimi et al 25 presented a high overall risk of bias, potentially due to methodological shortcomings or issues relating to reporting or data integrity, necessitating caution when interpreting its findings. The studies by Al-hebshi et al 20 and Alshahrani et al 21 raised some concerns regarding bias. While these studies may still offer valuable insights, these concerns indicate that certain aspects of their design or conduct may have introduced potential sources of bias, which should be considered when interpreting the results. The overall risk of bias in the studies by Al Dosari et al 19 and Wyne 33 remained unclear.

### Demographic Variables Analyzed

In the study by Al Agili and Alaki, 16 the focus was on males aged 9 and 14, with a larger sample size of males (875) compared to females (780). A similar study by Al Agili and Park 17 focused on the age range of 13 to 20 years, exclusively gathering data from male subjects (270). Al-Banyan et al 18 evaluated both male (154) and female (118) subjects within the age range of 5 to 12 years. Al Dosari et al 19 conducted an assessment with no gender ratio provided, focusing on ages 6 to 7 and 12 to 13. Al-hebshi et al 20 assessed children aged between 6 and 12 years, including both males (142) and females (128). Alshahrani et al 21 targeted older adolescents aged 15 to 17 years, with a significant male-only sample size of 3,411. Subsequent research by Al Zahidy, 22 Alghamdi and Almahdy, 23 and Alhammad and Wyne 24 had varying age ranges and gender ratios. Alkarimi et al 25 and Alshehhi et al 27 also provided their findings, with slight variations in the age groups and gender ratios. Additional evidence was offered by Aqeeli et al, 28 Bhayat and Ahmad, 29 Farooqi et al, 30 and Farsi, 31 each study focusing on specific age ranges and providing gender-specific data. The most recent study by Orfali et al 32 and an earlier study by Wyne 33 further enriched the body of evidence, each with large sample sizes and specific age group focuses.

### Inferences Assessed

Al Agili and Alaki 16 reported a high percentage of caries (73.42%), however, the corresponding DMFT index was not specified. Similarly, Al Zahidy 22 and Wyne 33 did not provide DMFT values but reported high caries percentages of 89.2 and 90.5%, respectively. Al Agili and Park 17 found a reduced caries prevalence (56%) and reported a DMFT index of 2.1 ± 2.77, suggesting a moderate level of dental DMFT. This was lower than the DMFT index reported by Al-Banyan et al 18 (3.8 ± 3.2) and Al Dosari et al 19 (5.06 ± 3.65), who found exceptionally high caries percentages of 99.3 and 91.2%, respectively. Al-hebshi et al 20 and Alshahrani et al 21 presented similar findings, with high caries percentages (93 and 72.9%, respectively) and moderate DMFT indices (1.98 ± 2.10 and 4.3 ± 5.59, respectively). In contrast, Alghamdi and Almahdy 23 reported a lower caries percentage (54.1%) and a relatively low DMFT index (1.26 ± 4.66). Alhammad and Wyne 24 reported a very high percentage of caries (98.6%), yet did not specify the corresponding DMFT index. On the other hand, Alkarimi et al 25 observed a remarkably low caries percentage (4.8%) and a high DMFT index (5.7 ± 4.2). al-Shammery, 26 Alshehhi et al, 27 Aqeeli et al, 28 Bhayat and Ahmad, 29 Farooqi et al, 30 and Farsi 31 all reported varied caries percentages and corresponding DMFT indices, contributing to a broad range of findings. Notably, the caries percentage (99.04%) and DMFT index (2.93 ± 2.29) reported by Farsi 31 were particularly high. The most recent study by Orfali et al 32 reported a caries percentage of 61.7% and a DMFT index of 2.42 ± 2.52, indicating a moderate level of dental DMFT.

### Caries Prevalence Analysis

**Fig. 3:**
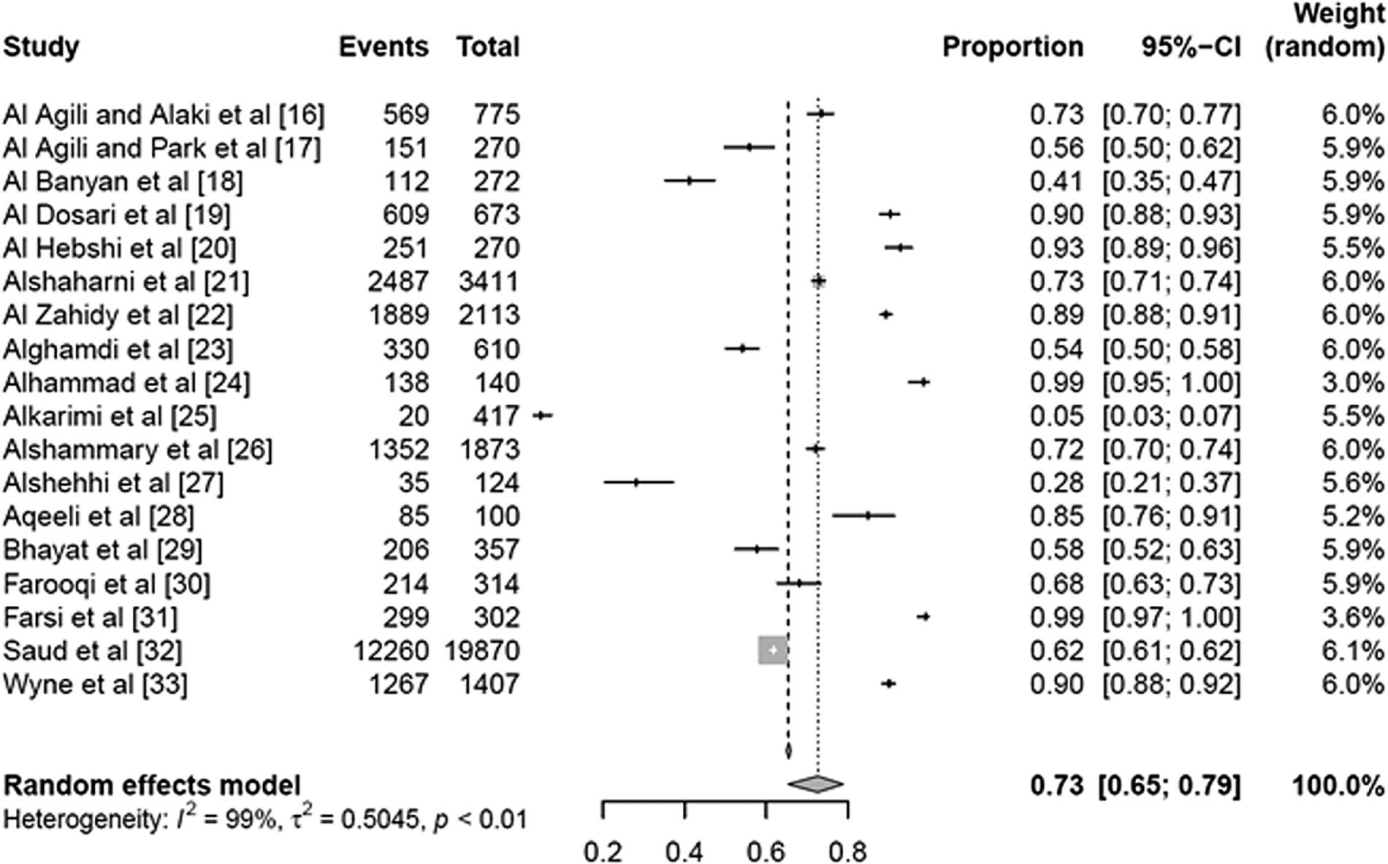
Prevalence of dental caries across the permanent teeth that were assessed across the studies.

As elucidated through Fig. 3 , the RE model estimated a higher caries prevalence rate of 72.62% (with a 95% CI of 65.3–78.89%). The heterogeneity statistics confirmed the significant variability among the included studies. The *I* ^2^ statistic was 99.0%, indicating that 99.0% of the total variability in the estimated effects (prevalence rates) was due to true variation in effects across studies rather than chance. This high level of heterogeneity was further confirmed by a large Q-statistic (1757.97) with 17 degrees of freedom, yielding a *p* -value of less than 0.0001, which indicates a statistically significant heterogeneity.

### Mean DMFT Analysis

**Fig. 4:**
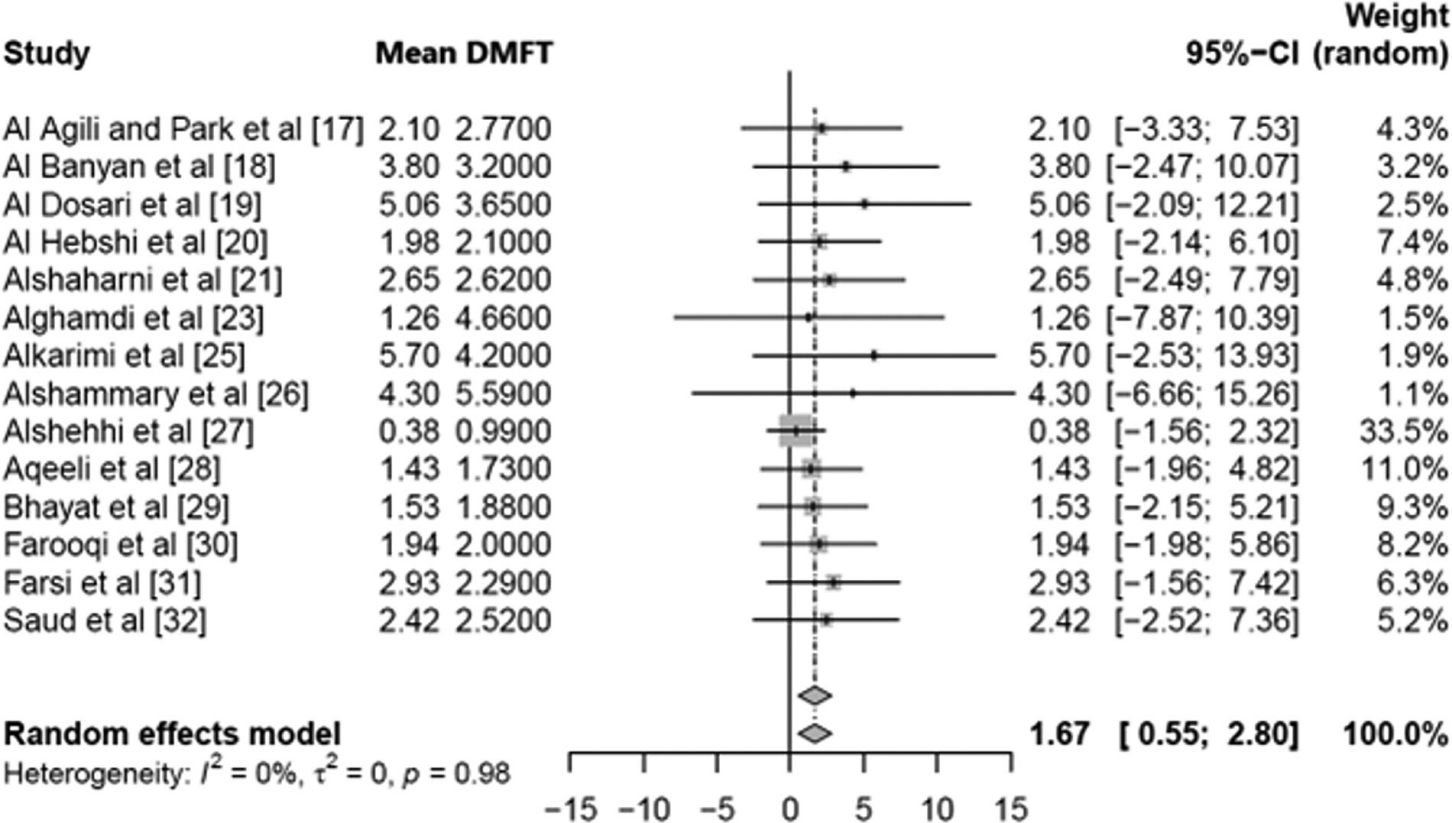
Aggregate effect approximations for the mean Decayed, Missing, and Filled Teeth (DMFT) scores assessed across the selected papers.

The analysis of the DMFT scores for permanent teeth across 14 studies is presented in Fig. 4 . The mean DMFT score was 1.675, with a 95% CI of 0.5519 to 2.7980. This result was statistically significant, as indicated by a *z* -score of 2.92 and a *p* -value of 0.0035, which is less than the conventional threshold of 0.05 for statistical significance. In terms of heterogeneity, the tau ^2^ value was 0, and the *I* ^2^ statistic was 0.0%, indicating no observed heterogeneity among the studies. This lack of heterogeneity was confirmed by the Q-statistic (4.77) with 13 degrees of freedom, resulting in a *p* -value of 0.9798. This high *p* -value suggests that the studies were homogeneous ( Table 2 ).

**Table 2:** Studies selected for the review and their observed assessments

Study	Year	Age range (in years) assessed	Gender ratio	Caries percentage	DMFT assessed
Al Agili and Alaki 16	2014	9, 14	Males - 875, Females- 780	73.42	NA
Al Agili and Park 17	2013	13–20	Males – 270	56	2.1 ± 2.77
Al-Banyan et al 18	2001	5–12	Males - 154, Females - 118	99.3	3.8 ± 3.2
Al Dosari et al 19	2004	6–7, 12–13	NA	91.2	5.06 ± 3.65
Al-hebshi et al 20	2015	6–12	Males - 142, Females - 128	93	1.98 ± 2.10
Alshahrani et al 21	2018	15–17	Males – 3,411	72.9	4.3 ± 5.59
Al Zahidy 22	2017	12–14	Males - 1,123, Females - 990	89.2	NA
Alghamdi and Almahdy 23	2017	14–16	Males - 610	54.1	1.26 ± 4.66
Alhammad and Wyne 24	2010	3–6, 7–9, 10–12	Males - 82, Females - 58	98.6	Unspecified
Alkarimi et al 25	2014	6–8	Males - 175, Females - 242	4.8	5.7 ± 4.2
al-Shammery 26	1999	12–13	Males - 937, Females - 937	68	2.65 ± 2.62
Alshehhi et al 27	2020	5–10	Males - 66, Females - 58	38.7	0.38 ± 0.99
Aqeeli et al 28	2021	9–12	Males - 601, Females - 399	85.1	1.43 ± 1.73
Bhayat and Ahmad 29	2014	12	Males - 357	57.2	1.53 ± 1.88
Farooqi et al 30	2015	6–12	Males – 406	68	1.94 ± 2.0
Farsi 31	2008	6–11, 12–17	Males - 179, Female - 133	99.04	2.93 ± 2.29
Orfali et al 32	2023	6, 12, 15	Males -10,435, Females - 9,435	61.7	2.42 ± 2.52
Wyne 33	2004	12–13	Males - 723, Females - 684	90.5	N/A

Abbreviations: DMFT, Decayed, Missing, and Filled Teeth; NA, not available.

## Discussion

The conclusions drawn for this study carry very important impacts for the general public health, especially in pediatric dental care within the Arab region. High prevalence in permanent teeth caries is 72.62%. In this regard, permanent teeth caries are considered to be a very significant load that forms within children. This figure, coupled with the moderate-to-high DMFT scores in the various research studies, reflects a naked need for adequate targeted interventional measures toward reducing the caries burden within the specified region. Since heterogeneity, as shown by *I* ^2^ of 0%, is missing in the DMFT analysis, a uniform degree of dental decay may be drawn uniformly across the region; thus, public health strategies within the region may need uniform approaches.

Other interesting findings from this research include the fact that there is a great variation in caries prevalence, as indicated by the high heterogeneity between caries rates within included studies ( *I* ^2^ = 99.0%). This variation may be representative of a difference in socioeconomic conditions, access to dental care, and the eating habits of those populations, as well as educational programs across the Arab world. Understanding this variation and regional difference is necessary for effective prevention and treatment strategies. There is also scope of potential areas left undetected by current monitoring systems for dental health. Partly, some studies did not report their DMFT in association with a high prevalence rate of caries. This limitation signifies that there should be standardized reporting practices in future research to ensure that comprehensive assessments of dental health are accomplished.

The study's findings, particularly the high prevalence of dental caries and the significant DMFT indices, underscore the critical oral health issues affecting the Arab child population. Al Agili and Alaki, 16 Al Zahidy, 22 and Wyne, 33 among others, reported substantial caries percentages. Although some studies did not specify the corresponding DMFT values, the high caries percentages alone highlighted the alarming state of oral health. Intriguingly, Al Agili and Park 17 found a relatively lower caries prevalence and a moderate DMFT index, revealing a degree of variation in the oral health status. Such differences might stem from multiple factors, such as geographical locations, socioeconomic conditions, or oral health practices, necessitating further investigation to pinpoint precise causes. The extreme caries percentages reported by Al-Banyan et al 18 and Al Dosari et al 19 were particularly alarming, indicating an urgent need for interventions. Similarly, the high caries percentages and moderate DMFT indices reported by Al-hebshi et al 20 and Alshahrani et al 21 underscored the pervasiveness of dental caries. In contrast, Alghamdi and Almahdy 23 reported a lower caries percentage, suggesting possible variations in oral health status across different regions or populations.

Our study, similarly to Khan 34 and Al Ayyan et al, 35 aimed to understand the prevalence and severity of dental caries. However, upon comparison, our results present unique characteristics and findings. Khan 34 reported a mean DMFT score of 4.341 for primary teeth and a DMFT score of 2.469 for permanent teeth among 2- to 20-year-olds in the Arab League. They observed considerable heterogeneity among the studies and acknowledged potential publication bias.

In contrast, Al Ayyan et al 35 analyzed dental caries prevalence in preschool children in the Gulf Cooperation Council area, reporting an overall mean DMFT in primary teeth of 5.14, with a prevalence of 80.9%. They also reported high heterogeneity in their selected studies, but unlike Khan, 34 they did not find evidence of publication bias. Our study presents a distinct prevalence of caries, indicating that patterns of dental caries can differ across populations due to variations in factors such as dietary habits, oral hygiene practices, and access to dental care.

The DMFT scores in our study differ from those reported by Khan 34 and Al Ayyan et al, 35 suggesting a unique level of dental caries severity in our study population. The high heterogeneity observed in both Khan 34 and Al Ayyan et al 35 is also present in our study, confirming that the effects of various factors on dental caries prevalence and severity vary across studies. Moreover, our study did not observe the same potential publication bias detected in the Khan 34 study, aligning more closely with the findings of Al Ayyan et al. 35 This suggests that our study and that of Al Ayyan et al 35 had a more evenly distributed set of research sources and methodologies.

In our study, we also focused on the prevalence of dental caries, similar to the studies conducted by Alshammari et al 36 and Adam et al. 37 Comparing our results with these studies, we find both similarities and differences that underscore the unique insights from our research. Alshammari et al 36 conducted a systematic review to understand the prevalence of dental caries in the Kingdom of Saudi Arabia. They identified 49 cross-sectional studies and reported that the proportion of dental caries in primary teeth ranged from 0.21 to 1.00, and in permanent teeth from 0.05 to 0.99. Adam et al 37 focused on children's dental health, conducting a meta-analysis to assess the prevalence and severity of dental caries among school children in Saudi Arabia. They extracted data from 18 studies, involving 56,327 children. The pooled estimate for the prevalence of caries among 5 to 7 years children was 84%, and among 12 to 15 years children it was 72%. In contrast to these findings, our study demonstrates distinct prevalence rates, which can be attributed to differences in the populations studied, age groups, dietary habits, oral hygiene practices, and access to dental care.

Our study reports a different range of caries prevalence in primary and permanent teeth than that found by Alshammari et al, 36 indicating a unique pattern of dental caries in our study population. Both the Alshammari et al 36 and Adam et al 37 studies exhibit high heterogeneity. Our study also shows high heterogeneity, confirming that the effects of various factors on dental caries prevalence vary across studies. The age groups studied by Adam et al 37 and the corresponding prevalence rates differ from those in our study. This discrepancy signifies that age is a significant factor influencing dental caries prevalence.

In the context of global dental health, several literature reviews have been conducted to understand the burden of caries in different countries. For instance, a comprehensive systematic review by Khan et al investigated the prevalence of dental caries in Saudi Arabia, focusing on articles published between 1999 and 2008. 2 The study unveiled a higher incidence of caries in primary teeth (mean DMFT score of 5.38) as compared to permanent teeth (mean DMFT score of 3.34). This pattern was corroborated in a separate study in another paper, 38 which examined trends in dental caries among children aged 5 to 6 years and 11 to 13 years in Latin American and Caribbean populations. They reported a greater prevalence of caries in primary teeth as compared to permanent teeth. Beyond prevalence, some reviews have delved into potential factors associated with dental caries. A notable study by Maupomé et al 39 explored the relationship between asthma and dental caries. Another line of research 40 41 has highlighted the potential risk associated with the consumption of soft drinks, which can provoke erosive effects on dental tissues due to their low pH levels (typically below 5.0–5.7). These studies underscore the multifactorial nature of dental caries, influenced by both systemic health conditions and lifestyle factors.

### Limitations

The study was subject to several limitations directly influencing the comprehensiveness and applicability of the findings. First, the lack of specified DMFT indices by Al Agili and Alaki, 16 Al Zahidy, 22 Wyne, 33 and Alhammad and Wyne 24 limited the depth of the comparative analysis. Without these DMFT indices, the association between caries percentage and the severity of dental DMFT could not be fully explored in these specific cases. Second, the considerable variation in both caries prevalence and DMFT indices across studies introduced significant heterogeneity into the data. This was evident in the substantial variability in the caries prevalence rates between the fixed effects and RE model, as well as the high *I* ^2^ statistic of 99.0%. Despite the use of the RE model to account for this variability, the high degree of heterogeneity remained a challenge in drawing definitive conclusions. Additionally, although the mean DMFT analysis revealed a significant level of dental DMFT, the absence of heterogeneity among the studies was somewhat unexpected given the considerable variation in caries percentages. This lack of observed heterogeneity could suggest that the DMFT indices might not have accurately reflected the severity of dental caries across different studies or populations.

### Recommendations

The high prevalence of dental caries and the associated DMFT index among children in the Arab region necessitates urgent action. A comprehensive, multipronged approach is needed to tackle this issue. One integral part of this approach is the promotion of regular dental checkups. By incorporating routine dental health assessments into school health programs, early detection and management of dental caries can become commonplace.

Furthermore, it is recommended that government bodies and relevant stakeholders implement comprehensive oral health programs. These programs should focus not only on providing treatment, but also on preventive measures such as oral health education, promotion of healthy dietary habits, and the application of fluoride programs. To effectively combat dental caries, access to dental care services must be improved, particularly in underprivileged areas. This could involve the establishment of additional dental clinics in these areas and making dental care services more affordable for all individuals.

In addition to improving access to care, public awareness on the importance of dental hygiene and the consequences of dental caries should be enhanced. This could be achieved through public awareness campaigns using various platforms such as schools, community centers, and media outlets. Moreover, it is crucial that health professionals, especially those working in schools and community health centers, receive adequate training on dental caries. They should be well equipped to provide basic dental care and educate children and parents about preventive measures. Moreover, ongoing research and monitoring are vital for tracking the effectiveness of implemented programs and strategies. This information can help refine these initiatives and shape more effective approaches to combat the high prevalence of dental caries. The success of these strategies will largely hinge on the collective efforts of governments, health professionals, schools, parents, and the children themselves.

## Conclusion

The data revealed an extremely high rate of dental caries, estimated at 72.62%. This signals a substantial public health burden, demanding quick response. The mean DMFT score throughout the studies was revealed to be 1.675, suggesting severe difficulties related to dental DMFT among the children in the Arab region. This emphasizes the substantial influence of dental caries on the children's dental health. However, despite the high caries frequency, the DMFT values revealed unexpectedly minimal variability among the studies. This could indicate that the DMFT indices might not properly represent the severity and variation of dental caries across different groups or research. This demands further research to develop the instruments for assessing dental health. Summarily speaking, the study emphasizes a high prevalence of dental caries and large DMFT scores in youngsters in the Arab region, pointing to an urgent public health issue. However, the variance across studies and inherent limitations of DMFT indices call for greater study to completely comprehend and treat the burden of dental caries in this demographic.
